# Myeloid-specific blockade of Notch signaling ameliorates nonalcoholic fatty liver disease in mice

**DOI:** 10.7150/ijbs.80122

**Published:** 2023-03-27

**Authors:** Jian Ding, Ming Xu, Wei Du, Zhi-Qiang Fang, Hao Xu, Jing-Jing Liu, Ping Song, Chen Xu, Zhi-Wen Li, Zhen-Sheng Yue, Yu-Wei Ling, Juan-Li Duan, Kai-Shan Tao, Fei He, Lin Wang

**Affiliations:** Department of Hepatobiliary Surgery, Xi-Jing Hospital, Fourth Military Medical University, Xi'an 710032, China.

**Keywords:** nonalcoholic fatty liver disease, macrophage, Notch signaling, RBP-J, exosomes, transcription factor decoy

## Abstract

**Rationale:** Macrophages play a central role in the development and progression of nonalcoholic fatty liver disease (NAFLD). Studies have shown that Notch signaling mediated by transcription factor recombination signal binding protein for immunoglobulin kappa J region (RBP-J), is implicated in macrophage activation and plasticity. Naturally, we asked whether Notch signaling in macrophages plays a role in NAFLD, whether regulating Notch signaling in macrophages could serve as a therapeutic strategy to treat NAFLD.

**Methods:** Immunofluorescence staining was used to detect the changes of macrophage Notch signaling in the livers of human patients with NAFLD and choline deficient amino acid-defined (CDAA) diet-fed mice. Lyz2-Cre RBP-J^flox^ or wild-type C57BL/6 male mice were fed with CDAA or high fat diet (HFD) to induce experimental steatohepatitis or steatosis, respectively. Liver histology examinations were performed using hematoxylin-eosin (H&E), Oil Red O staining, Sirius red staining and immunohistochemistry staining for F4/80, Col1α1 and αSMA. The expression of inflammatory factors, fibrosis or lipid metabolism associated genes were evaluated by quantitative reverse transcription (qRT)-PCR, Western blot or enzyme-linked immunosorbent assay (ELISA). The mRNA expression of liver samples was profiled by using RNA-seq. A hairpin-type decoy oligodeoxynucleotides (ODNs) for transcription factor RBP-J was loaded into bEnd.3-derived exosomes by electroporating. Mice with experimental NAFLD were treated with exosomes loading RBP-J decoy ODNs via tail vein injection. *In vivo* distribution of exosomes was analyzed by fluorescence labeling and imaging.

**Results:** The results showed that Notch signaling was activated in hepatic macrophages in human with NAFLD or in CDAA-fed mice. Myeloid-specific RBP-J deficiency decreased the expression of inflammatory factors interleukin-1 beta (IL1β) and tumor necrosis factor alpha (TNFα), attenuated experimental steatohepatitis in mice. Furthermore, we found that Notch blockade attenuated lipid accumulation in hepatocytes by inhibiting the expression of IL1β and TNFα in macrophages *in vitro*. Meanwhile, we observed that tail vein-injected exosomes were mainly taken up by hepatic macrophages in mice with steatohepatitis. RBP-J decoy ODNs delivered by exosomes could efficiently inhibit Notch signaling in hepatic macrophages *in vivo* and ameliorate steatohepatitis or steatosis in CDAA or HFD mice, respectively.

**Conclusions:** Combined, macrophage RBP-J promotes the progression of NAFLD at least partially through regulating the expression of pro-inflammatory cytokines IL1β and TNFα. Infusion of exosomes loaded with RBP-J decoy ODNs might be a promising therapy to treat NAFLD.

## Introduction

Nonalcoholic fatty liver disease (NAFLD), has become the most common type of chronic liver disease, affecting more than a quarter of the world's population 1-3. NAFLD varies from hepatic steatosis characterized by accumulation of triglycerides in hepatocytes to nonalcoholic steatohepatitis (NASH), which might progress to fibrosis, cirrhosis and hepatocellular carcinoma 1-3. However, the molecular mechanisms of NAFLD has not been fully understood, and there are still limited appropriate drugs specifically to treat NAFLD.

Hepatic macrophages, the largest population of innate immune cells in the liver, which account for about 80% of the total macrophages in the body 4, 5. Hepatic macrophages play a critical role in the maintenance of liver homeostasis and in the development of liver disease, including NAFLD 6-8. There are two known functional subsets of macrophages: classically activated “pro-inflammatory” M1 macrophages and alternatively activated “anti-inflammatory” M2 macrophages 9, 10. In NAFLD, macrophages of a pro-inflammatory phenotype seem to contribute to disease severity 7, 10. High-fat diet (HFD)-fed mice had increased numbers of liver macrophages with preponderant production of pro-inflammatory cytokines 11. Zhang et al. 12 found that macrophage p38α causes M1 polarization, and deficiency of macrophage p38α attenuates experimental steatohepatitis in mice. On the other hand, M2 macrophages with an anti-inflammatory phenotype have been associated with attenuated hepatic injury and insulin resistance in NAFLD 11, 13. These findings indicate that the development of NAFLD are closely related to the activation of macrophages, especially M1 macrophages, and regulating macrophage activation may be an important strategy for the treatment of NAFLD.

Notch signaling mediated by RBP-J, the transcription factor transactivated by signals from four mammalian Notch receptors 14, is implicated in macrophage activation 15-17. Notch signaling is required for lipopolysaccharide (LPS)-stimulated macrophage activation through interferon regulatory factor 8 (IRF-8) [17]and nuclear factor ĸ-light-chain-enhancer of activated B cells (NF-ĸB) 18. We have previously shown that Notch blockade in macrophages could impair the expression of inflammatory factors interleukin-1 beta (IL1β) and tumor necrosis factor alpha (TNFα) by up-regulation of cylindromatosis (Cyld), and then ameliorate hepatic fibrosis in mice 19. Therefore, we speculated that inhibition of Notch signaling in macrophages may ameliorate NAFLD.

In this study, we showed that myeloid-specific RBP-J deficiency attenuated experimental steatohepatitis in mice, and the infusion of exosomes loaded with RBP-J decoy ODNs could represent a promising therapeutic strategy for the treatment of NAFLD.

## Materials and Methods

### Mice

Mice were maintained in a specific pathogen-free (SPF) condition on the C57BL/6 background. Lyz2-Cre RBP-J^flox^ mice were donated by Professor Han in Fourth Military Medical University. Littermates (Lyz2-Cre RBP-J^flox/+^) were used as the control of Lyz2-Cre RBP-J^flox/flox^ mice (RBP-J KO). C57BL/6 wild type mice were purchased from Gempharmatech Co., Ltd. (Nanjing, China). To induce experimental NAFLD, male mice (7-8 weeks of age) were fed with choline deficient amino acid-defined diet (CDAA, L-amino acid diet with 45% kcal% fat with 0.1% methionine no added choline, Research Diets, New Brunswick, NJ, USA) for 10 weeks, or high-fat diet (HFD, rodent diet with 60 kcal% fat, Research Diets) for 22 weeks. All animal experiments were performed following the guidelines of the Animal Experiment Administration Committee of Fourth Military Medical University.

### Human liver samples

Paraffin embedded liver tissues were obtained from the Department of Hepatobiliary Surgery of Xijing Hospital, Fourth Military Medical University. Liver samples were obtained from seven patients with NASH. Six control individuals had no history of diabetes, alcohol use, or viral hepatitis. All participants had signed informed consents for the use of their samples. The basic information of the patients is listed in Table S1. The protocols involving human samples were approved by the Ethics Committee of Xijing Hospital, Fourth Military Medical University.

### Histological analysis

Liver specimens were fixed with 10% buffered formalin or 4% paraformaldehyde (PFA). Formalin-fixed specimens were embedded in paraffin, sectioned, and routinely stained with hematoxylin and eosin (H&E). PFA-fixed samples were embedded with the compound at the optimal cutting temperature (OCT) and sectioned at 8-μm thickness. The presence of steatosis was confirmed using oil red O staining frozen liver sections. Liver sections were stained with freshly prepared saturated oil red O solution (Servicebio, Wuhan, China) according to standard procedures. Immunofluorescence (IF) and immunohistochemical (IHC) staining were described previously 20.

### Biochemistry and Enzyme-linked immunosorbent assay (ELISA)

Serum albumin, alanine aminotransferase (ALT), aspartate aminotransferase (AST), low density lipoprotein (LDL), and triglyceride (TG) levels were determined using an automatic biochemical analyzer (Rayto Life and Analytical Sciences Company, Shenzhen, China).

The levels of IL1β and TNFα in serum or culture supernatants were measured using kits from Thermo Fisher Scientific (MA, USA) following the manufacturer's protocols.

### Cell culture

Mouse brain endothelial cell line bEnd.3 cells or RAW264.7 macrophages were cultured in DMEM supplemented with 10% fetal bovine serum (FBS), 2 mM L-glutamine, 100 U/ml penicillin and 100 μg/ml streptomycin at 37°C in a 5% CO_2_ incubator. AML12 hepatocytes were cultured in DMEM/F-12 (1:1) medium supplemented with 10% FBS, 1% ITS Liquid Media Supplement (Sigma, I3146), Dexamethasone (40 ng/ml) and antibiotics.

Hepatic macrophages were isolated by using magnetic bead cell sorting (MACS) as described 19,20. Hepatic macrophages cultured with the fresh medium containing lipopolysaccharide (LPS, 100 ng/ml) for 24 h. RAW264.7 macrophages were treated with γ-secretase inhibitor N-[N-(3,5-Difluorophenacetyl)-L-alanyl]-S-phenylglycine t-butyl Ester (DAPT, 5 μM, Selleck Biotechnology Co. LTD, USA) or DMSO for 72 h, changed and cultured with the fresh medium containing LPS for 24 h, and the conditioned medium (CM) were harvested. Next, AML12 hepatocytes were cultured in palmitic acid (PA, 10 mM, Kunchuang Science and Technology Development Co. LTD, Xi'an) medium with CM for 48 h. Then AML12 cells were stained with oil red O (Servicebio). Triglyceride content in AML12 cells was measured using a detection kit (Pulai Biological Co. LTD, Beijing) following the manufacturer's protocols.

### Isolation, identification and labeling of Exosomes

Mouse endothelial cell line bEnd.3 was cultured in FBS-free medium for 48 hours. Exosomes in supernatants were isolated using PEG6000 (Sigma) as described 20, 21. The size distribution of exosomes was analyzed by Laser Scattering Microscopy for nanoparticle tracking analysis (NTA), and the morphology of exosomes was observed by transmission electron microscopy (TEM) in Dolaimi Biotechnology Co., Ltd. (Wuhan, China).

To label exosomes, exosomes in PBS were incubated with DiI (Invitrogen) for 30 min at 37 ℃ according to the protocol, then washed and centrifuged with PBS containing 12% PEG6000 at 12000 × g for 30 min.

### Western blot

Liver tissues, bEnd.3 cells or exosomes were lysed with radioimmunoprecipitation assay (RIPA) buffer (Beyotime) containing phenylmethylsulfonyl fluoride (PSMF, 10 mM), and the protein concentration was quantified using the BCA Protein Assay Kit (Thermo Fisher Scientific, Rockford, IL, USA) following the manufacturer's instructions. The samples were analyzed by sodium dodecyl sulfate-polyacrylamide gel electrophoresis (SDS-PAGE), transferred to polyvinylidene fluoride (PVDF) membranes (Millipore, Billerica, MA, USA), and incubated with primary antibodies targeting IL1β, TNFα, CD9, Alix, Flotillin-1, and VDAC1 and then with HRP-conjugated goat anti-mouse or anti-rabbit IgG secondary antibodies. The information of the antibodies used is listed in Table S2.

### RBP-J decoy ODN loading and exosome injection

We have reported a hairpin RBP-J decoy ODNs in reference 20. In brief, two RBP-J binding sites (CGTGGGAA 22) were present in this decoy, with three phosphorothioatemodified sites at each end, whereas the control decoy was introduced mutation in the RBP-J binding sites. The sequence of RBP-J decoy ODNs was 5′-CTGCGTGGGAACTAGCGTGGGAATATTTTTTATATTCCCACGCTAGTTCCCACGCAG-3′; the sequence of the control decoy ODNs was 5′-CTGCGTTTTAACTAGCGTTTTAATATTTTTTATATTAAAACGCTAGTTAAAACGCAG-3′. All the ODNs were synthesized by Tsingke Biotechnology Co. Ltd. (Beijing, China). The decoy ODNs were resuspended at a concentration of 20 μM, incubated for 10 minutes at 95℃, then cooled down to room temperature slowly.

Exosomes were electroporated with decoy ODNs at 400 V and 125 μF in 0.4-cm electroporation cuvettes as previously described 20, 23.

For *in vivo* exosome tracking, CDAA-fed mice were injected with DiI-labeled exosomes via the tail vein. Tissues were harvested 6 h after injection for bioluminescence imaging (Andor iKon Dw434, Oxford Instruments, Oxon, UK) or tissue sectioning.

For exosome-mediated therapy, exosomes were loaded with decoy ODNs and infused into CDAA-fed mice in the 9^th^ and 10^th^ week or those with HFD-fed mice in 21^st^ and 22^nd^ week four times by tail vein injection. Mice were humanely euthanized for further analysis 2-3 days after the last exosome injection.

### RNA extraction and quantitative reverse transcription PCR (qRT-PCR)

Total RNA was isolated from liver tissues, hepatic macrophages, RAW264.7 macrophages or AML12 hepatocytes with Trizol reagent (Invitrogen) according to the manufacturer's instructions. The mRNA was reverse transcribed into cDNA using the Evo M-MLV RT Premix (Accurate Biology, China). qRT-PCR was performed with the SYBR Green Premix Pro Taq HS qPCR Kit (Accurate Biology, China) in a Bio -Rad CFX Maestro 2.2 Real-Time PCR System (Bio-Rad, Hercules, CA, USA). β-actin or GAPDH was served as the internal control. All primers were purchased from Tsingke Biotechnology Co. Ltd. (Beijing, China). The sequences of the primers used are listed in Table S3.

### Statistical analysis

Data were analyzed with Graph Pad Prism software, version 8.0. Comparisons between groups were undertaken using unpaired or paired Student's t-test. Some data that did not comply with normal distribution were analyzed by the Mann-Whitney test. The number of repetitions were indicated in the legends of each graph. Results were expressed as means ± SD. *P* < 0.05 was considered as significant.

## Results

### Notch signaling was activated in hepatic macrophages in human with NASH or in CDAA-fed mice

**Figure 1:**
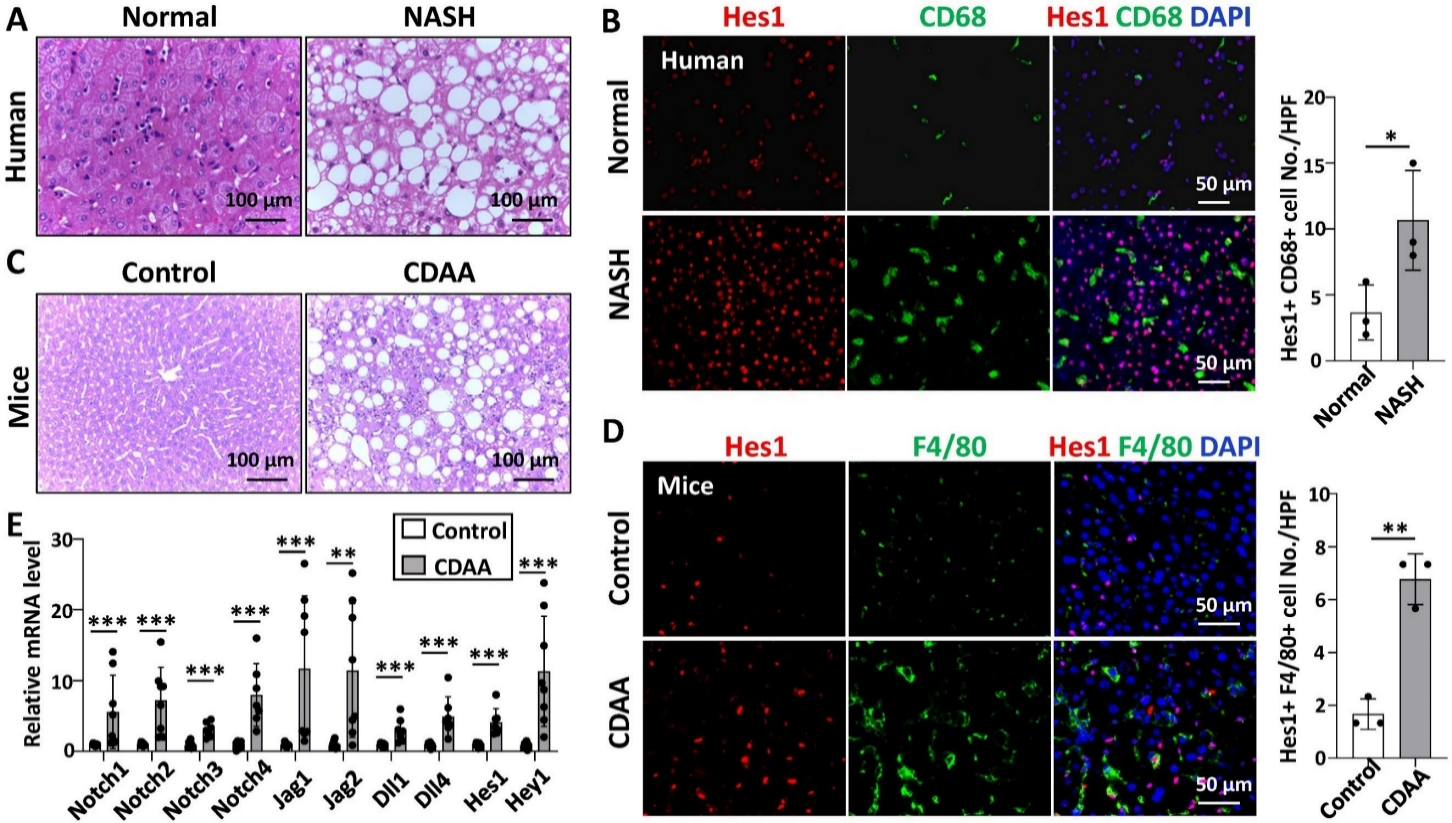
**Notch signaling in hepatic macrophages was activated in human with NASH or in CDAA-fed mice.** (A) H&E staining of liver tissues from normal or patients with NASH. (B) Immunofluorescence staining for Hes1 and macrophage marker CD68 in liver tissues from normal or patients with NASH, nuclei were counterstained with DAPI, and Hes1^+^ CD68^+^ were quantified and compared. (C) H&E staining of liver tissues in chow-fed or CDAA-fed mice. (D) Immunofluorescence staining for Hes1 and F4/80 in liver tissues from chow-fed or CDAA-fed mice, and Hes1^+^ F4/80^+^ were quantified. (E) The mRNA levels of Notch ligands, Notch receptors, and downstream genes in liver extracts from chow-fed or CDAA-fed mice were determined by qRT-PCR. Bars = means ± SD; * P < 0.05, ** P < 0.01, *** P < 0.001.

We examined the expression of Hes1, a key downstream gene of Notch signaling 14, in the liver tissues of patients with NASH and normal controls. As determined by Western blot and immunofluorescence staining, Hes1 expression was significantly higher in patients with NASH than in normal controls, and CD68^+^ hepatic macrophages mainly co-located with Hes1^+^ cells on liver sections (Fig. S1A,B, Fig. 1B).

We then detected the expression of Notch signaling related genes in livers of wild-type mice fed with CDAA for 10 weeks by qRT-PCR. The mRNA levels of almost all Notch ligands, Notch receptors, target genes Hes1 and Hey1 were significantly up-regulated in liver samples of CDAA-fed mice (Fig. 1E). The protein level of Hes1 was also significantly higher in the livers of CDAA-fed mice than chow-fed mice (Fig. S1C,D). Immunofluorescence staining further showed that Hes1 was up-regulated and co-localized with F4/80^+^ hepatic macrophages in the livers of CDAA-mice (Fig. 1D). These results suggested that Notch signaling was activated in hepatic macrophages during NAFLD progression.

### Myeloid-specific RBP-J deficiency attenuated experimental steatohepatitis in CDAA-fed mice

We investigated the role of macrophage Notch signaling in the development of steatohepatitis. RBP-J*^flox^* mice were crossed with Lyz2-Cre transgenic mice to obtain myeloid-specific RBP-J knockout (KO) and control mice. The efficiency of RBP-J deletion in liver macrophages was confirmed by qRT-PCR of genomic DNA as described 19. Myeloid-specific RBP-J KO and control mice were fed with CDAA diet for 10 weeks.

**Figure 2:**
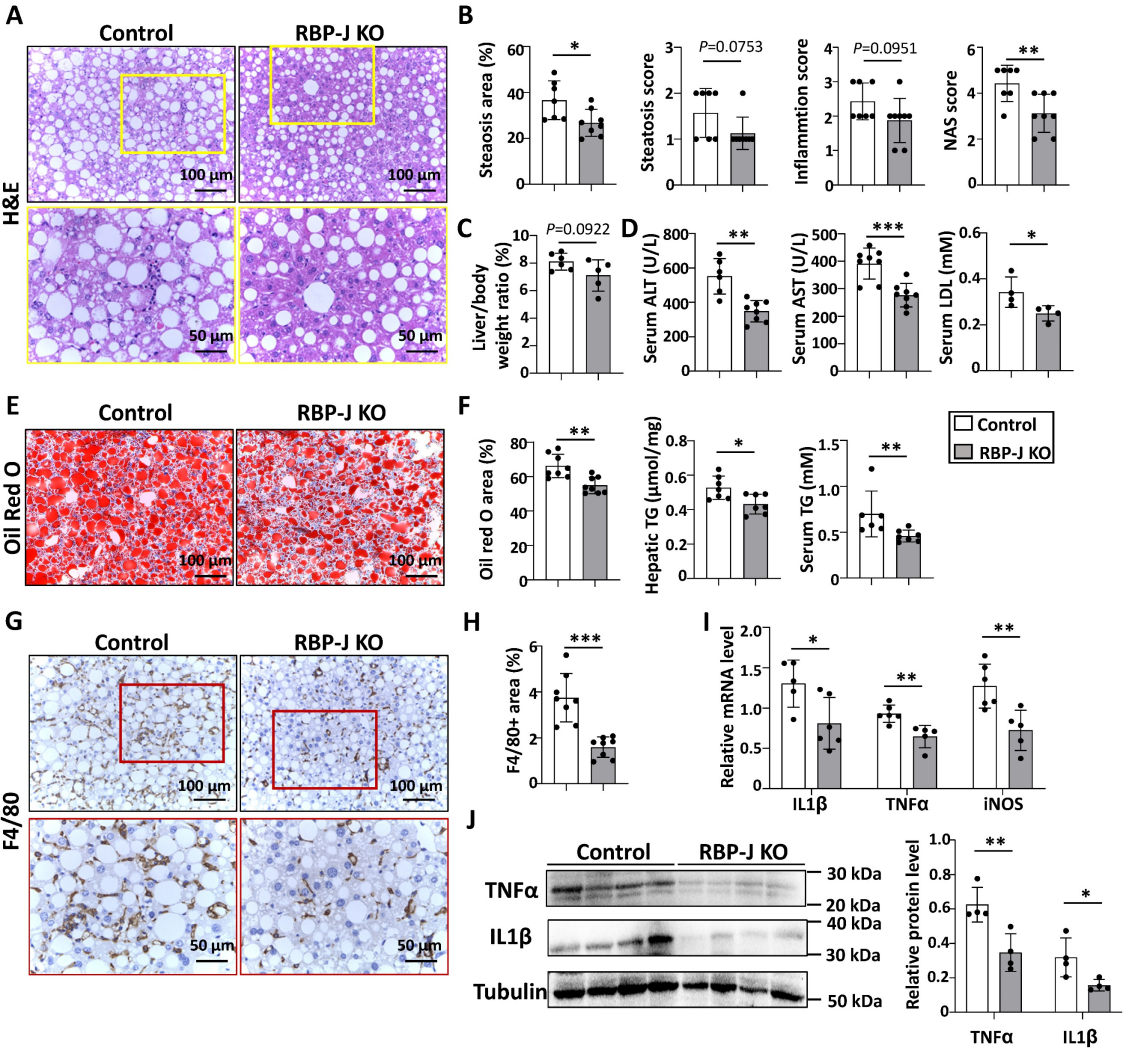
** Myeloid-specific RBP-J deficiency attenuated experimental steatohepatitis in CDAA-fed mice.** Lyz2-Cre^+^ RBP-J^flox/+^ (Control) or Lyz2-Cre^+^ RBP-J^flox/flox^ (KO) mice were fed with CDAA diet for 10 weeks. (A) Liver sections were stained by H&E staining. The lower row of micrographs were a higher magnification of the yellow frames in the upper row. (B) Quantitative comparison of steatosis areas, and histological scores about steatosis, inflammation and NAS in (A). (C) Liver-to-body weight ratio. (D) Detection of ALT, AST and LDL in serum. (E) Liver sections were stained with Oil Red O. (F) Quantitative comparison of positive signals for Oil Red O in (E), and detection of triglyceride content in livers and serum. (G) Liver sections were subjected to immunohistochemical staining for F4/80. The lower row of micrographs were a higher magnification of the red frames in the upper row. (H) The positive areas of F4/80 staining in (G) were quantitatively compared. (I) The mRNA levels of IL1β, TNFα and iNOS in livers were determined by qRT-PCR. (J) The protein levels of IL1β and TNFα were determined by Western blot, with Tubulin as a reference control. Bars = means ± SD; * P < 0.05, ** P < 0.01, *** P < 0.001.

The serum ALT, AST and LDL were significantly decreased compared with control mice (Fig. 2D), suggesting ameliorated liver damage in myeloid-specific RBP-J KO mice. Hematoxylin and eosin (H&E) staining showed fewer large round non-staining areas (represent lipid droplets in hepatocytes) and inflammatory cells, and lower NAS score in livers from RBP-J KO mice compared with control mice (Fig. 2A,2B). Oil red O staining of liver sections showed that RBP-J KO group had less lipid droplets compared with control group (Fig. 2E,2F). In keeping with this, triglyceride content in serum or liver was significantly decreased in RBP-J KO mice as compared with the control mice (Fig. 2F).

Immunostaining with anti-F4/80 and anti-MPO showed that the infiltration of macrophages and neutrophils was less in livers from RBP-J KO mice (Fig. 2G,2H, Fig. S2). Accordingly, the levels of inflammatory mediators were determined using qRT-PCR and Western blot. The results indicated that IL1β, TNFα and iNOS were decreased in the livers from RBP-J KO mice (Figure 2I,2J). The level of IL1β in the serum also decreased in RBP-J KO mice as compared with the control mice (Fig. S3A).

Furthermore, we evaluated hepatic fibrosis by anti-Collagen Type I Alpha 1 Chain (Col1α1), anti-Actin Alpha 2, Smooth Muscle (αSMA) immunostaining and Sirius red staining. RBP-J KO mice displayed significantly reduced extracellular matrix (ECM) deposition and activation of hepatic stellate cells (Fig. S4A,S4B). Consistently, the mRNA levels of tissue inhibitor of metalloproteinases 1/2 (TIMP1/2), Col1α1 and αSMA decreased in the livers of CDAA-fed RBP-J KO mice, while the mRNA level of matrix metallopeptidase 9 (MMP9) increased (Fig. S4C). These results indicated that RBP-J deficiency in macrophages ameliorated steatohepatitis and steatohepatitis-induced fibrosis in mice.

### Notch blockade impaired the expression of IL1β and TNFα in macrophages, and then attenuated lipid accumulation in hepatocytes *in vitro*

Next, we explored the possible mechanism of Notch blockade in macrophages affecting lipid metabolism in hepatocytes. The pro-inflammatory cytokines IL1β and TNFα can increase lipid accumulation in hepatocytes 12, 24-26. Meanwhile, our previous studies found that Notch blockade significantly reduced the expression of IL1β and TNFα in macrophages 19. Therefore, we speculate that Notch-IL1β/TNFα axis could play a critical role during the activation of macrophages and lipid accumulation in hepatocytes.

**Figure 3:**
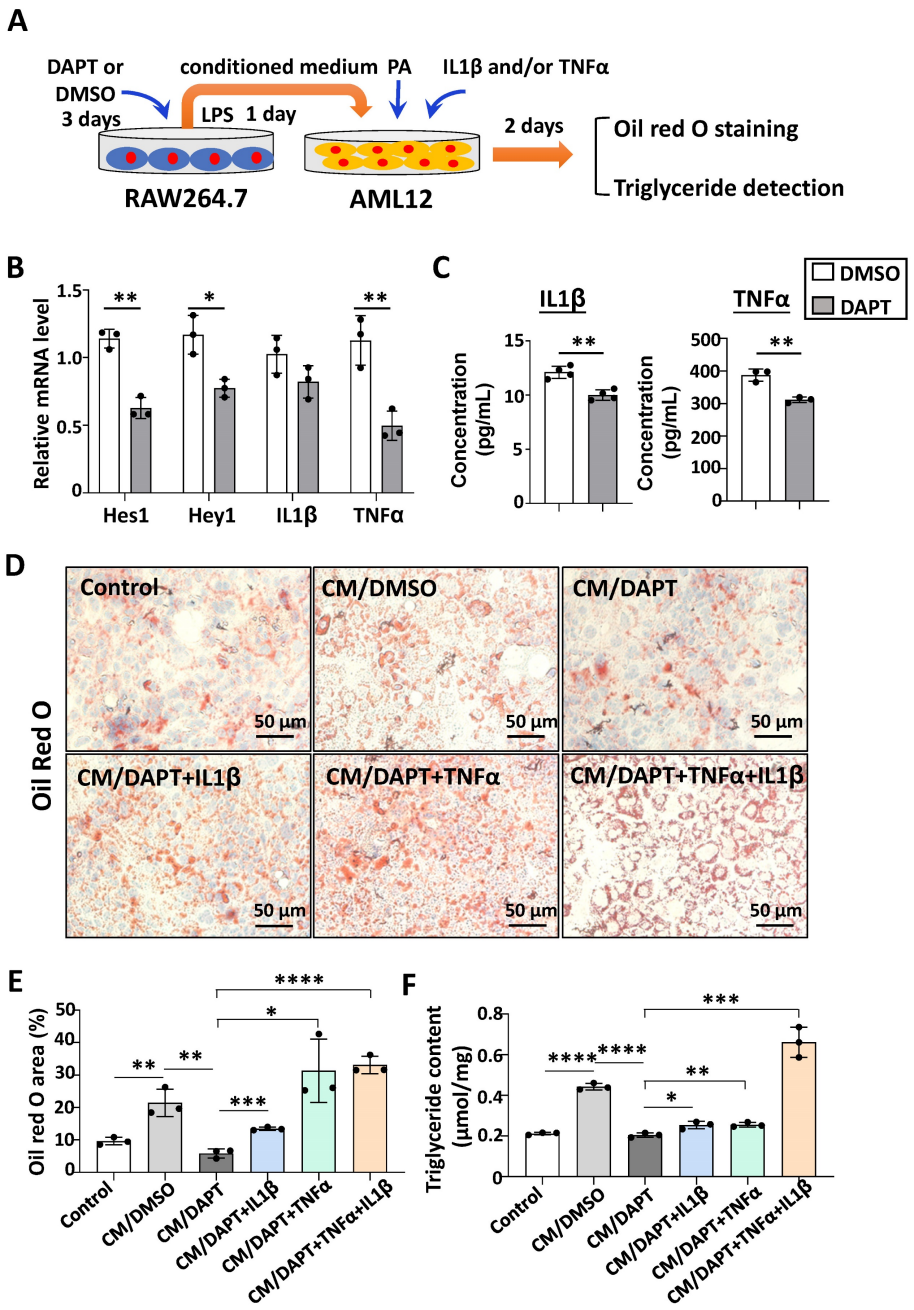
** Notch blockade in macrophages attenuated lipid accumulation in hepatocytes through inhibiting the expression of IL1β and TNFα *in vitro*.** (A) Schematic showing that RAW264.7 macrophages were treated with γ-secretase inhibitor DAPT (5 μM) for 3 days, changed and cultured with the fresh medium containing LPS (100 ng/ml) for 1 day, and the conditioned medium (CM) were harvested. Then AML12 hepatocytes were cultured with CM, in the presence or absence of TNFα (10 ng/ml), and/or IL1β (10 ng/ml) in palmitic acid medium (PA, 10 mM) for 2 days. (B) The mRNA levels of Hes1, Hey1, IL1β and TNFα in RAW264.7 macrophages treated with DMSO or DAPT were determined by qRT-PCR. (C) The levels of IL1β and TNFα in RAW264.7 culture supernatants were measured by ELISA. (D) Lipid accumulation in AML12 cells was assessed with Oil Red O staining. (E) The positive areas of Oil Red O staining in (D) were quantitatively compared. (F) Triglyceride content in AML12 hepatocytes was measured. Bars = means ± SD; * P < 0.05, ** P < 0.01, *** P < 0.001, **** P < 0.0001.

RAW264.7 macrophages were treated with γ-secretase inhibitor DAPT, an inhibitor for Notch signaling, for 3 days. The mRNA levels of Notch target genes Hes1 and Hey1 decreased (Fig. 3B), suggesting that Notch signaling was inhibited successfully. Meanwhile, the expression of IL1β and TNFα were determined at the mRNA and protein levels by using qRT-PCR and ELISA, respectively (Fig. 3B,3C). The results showed that the production of these inflammatory cytokines decreased significantly in DAPT-treated RAW264.7 as compared with the control. Then AML12 hepatocytes were cultured in palmitic acid medium (PA) with conditioned medium (CM) collected from each of the cultures of RAW264.7 for 2 days. We evaluated the lipid accumulation in AML12 hepatocytes by oil red O staining and triglyceride content assay. As shown in Fig. 3D-F, CM derived from DAPT-treated RAW264.7 showed reduced capacity of inducing lipid accumulation in AML12 hepatocytes, but this capacity was rescued partially by supplementing IL1β or TNFα, and recovered completely by supplementing IL1β and TNFα. Meanwhile, AML12 hepatocytes were also cultured with CM derived from hepatic macrophages. As shown in Fig. S5, CM derived from RBP-J KO macrophages reduced lipid accumulation in AML12 hepatocytes, but this capacity was rescued by supplementing IL1β or/and TNFα. Collectively, these results suggested that Notch blockade in macrophages inhibited the secretion of pro-inflammatory cytokines IL1β and TNFα, and then ameliorated lipid accumulation in hepatocytes.

### Myeloid-specific RBP-J deficiency up-regulated the expression of genes related to fatty acid degradation and peroxisomal fatty acid oxidation in livers of CDAA-fed mice

To explain how pro-inflammatory cytokines IL1β and TNFα affect lipid accumulation in hepatocytes, we compared mRNA profiles of liver tissues between CDAA-fed RBP-J KO and control mice by using RNA-seq (Fig. S6A). Gene set enrichment analyses showed that fatty acid degradation, peroxisome, fatty acid metabolism and peroxisome proliferator activated receptor (Ppar) signaling pathway associated genes were up-regulated in RBP-J KO group (Fig. S6B). Several genes associated fatty acid metabolism, including Pparɑ, cytochrome P450 family 4 subfamily a member 12a (Cyp4a12a), Cyp4a12b, acetyl-CoA acyltransferase 1a (Acaa1a), nudix hydrolase 7 (Nudt7) and peroxisomal biogenesis factor 11 alpha (Pex11ɑ), attracted our attention. As confirmed by qRT-PCR, the expression of Pparɑ, Cyp4a12a, Cyp4a12b, Acaa1a, Nudt7 and Pex11ɑ were up-regulated in RBP-J KO group (Fig. S6C). Studies have shown that Cyp4a12a/b is associated with fatty acid degradation 27,28, and Acaa1a, Nudt7, Pex11a are related to peroxisomal fatty acid oxidation 29-34. Pparɑ could regulate the expression of Cyp4a12a/b, Acaa1a and Pex11ɑ 27-30,34. Knockout of Acaa1a, Nudt7 or Pex11ɑ promoted hepatic steatosis 30-33. Meanwhile, hepatic macrophages promoted hepatic steatosis via IL1β-dependent suppression of Pparɑ activity 24. Loft et al. also found that TNFɑ expressed by macrophages could affect the activity of Pparɑ in hepatocytes and inhibit the expression of Pparɑ downstream genes 35. Therefore, we speculated that RBP-J knockout in macrophages reduced the expression of IL1β and TNFɑ, and these reduced inflammatory factors could increase the expression or activity of Pparɑ in hepatocytes and then up-regulate the expression of genes related to fatty acid degradation and peroxisomal fatty acid oxidation, which ultimately would reduce fat accumulation.

### Characterization of exosomes derived from bEnd.3 cells

We examined the effects of Notch inhibition in macrophages on the development of steatohepatitis. In our previous work, we demonstrated that exosomes loaded with RBP-J decoy could inhibit Notch signaling in hepatic macrophages and ameliorate CCl_4_- or BDL-induced liver fibrosis in mice 20. It is natural to ask whether this therapeutic strategy can be extended to steatohepatitis.

**Figure 4:**
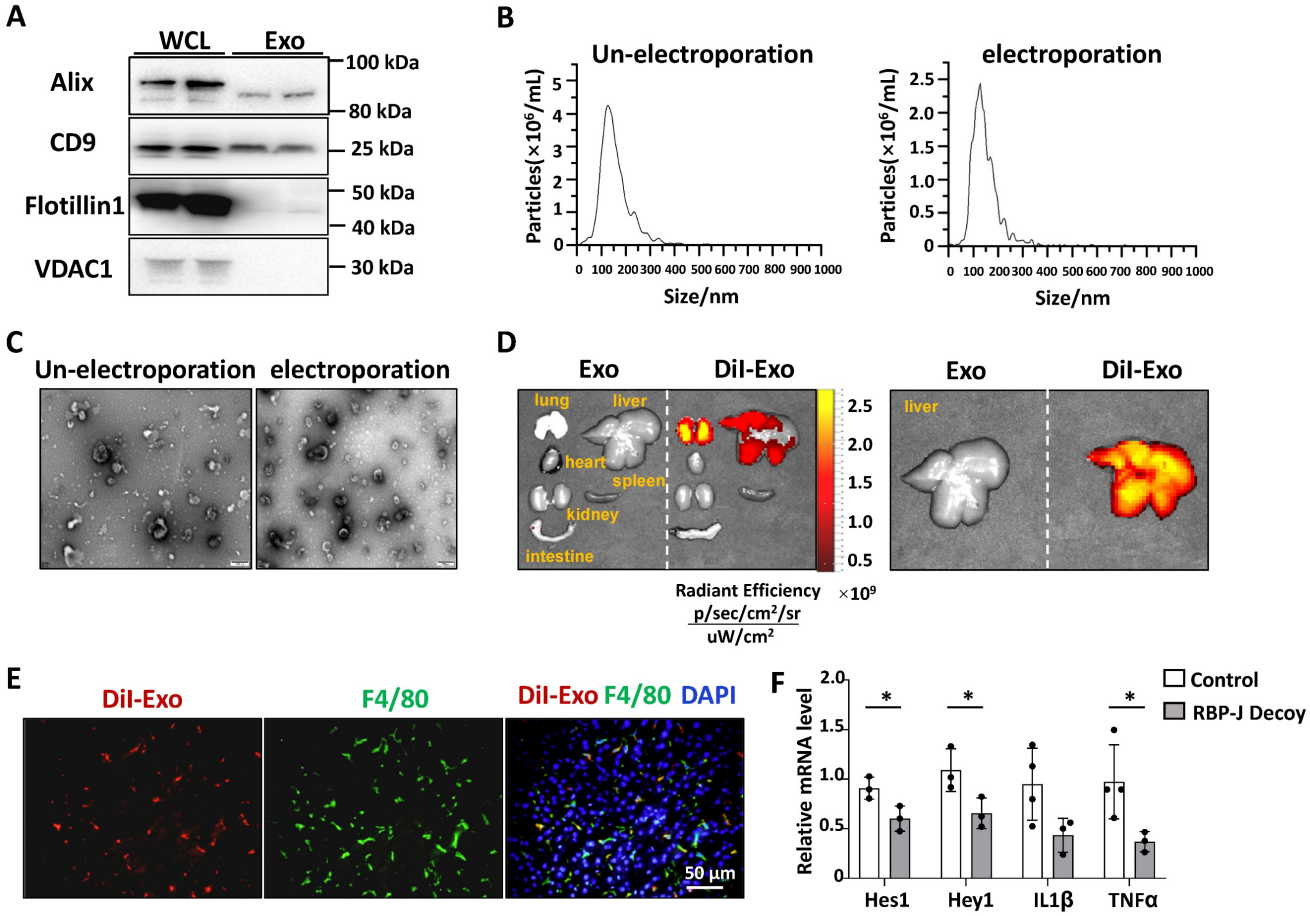
** Exosomes loaded with RBP-J decoy oligodeoxynucleotide (ODNs) inhibited Notch signaling in hepatic macrophages in a murine model of steatohepatitis.** (A-C) Characterization of exosomes derived from bEnd.3 cells. (A) The levels of CD9, Alix, flotillin-1, and VDAC1 in exosomes and bEnd.3 cells lysates were determined by western blot. (B) The size distribution of exosomes was determined by nanoparticle tracking analysis (NTA). (C) Exosome morphology was observed by transmission electron microscopy (TEM). (D) Exosomes were stained with DiI and approximately 200 µg (protein equivalent) of exosomes in 150 µL of PBS were injected via the tail vein into mice with CDAA-induced steatohepatitis. After 6 h, DiI signals in the liver, lung, spleen, intestine, kidney, and heart were examined using bioluminescence imaging. (E) 6 h after injection of DiI-labeled exosomes, liver sections were stained with an anti-mouse F4/80 antibody and analyzed by fluorescence microscopy. Nuclei were counterstained with DAPI. (F) Mice were fed with CDAA diet for 10 weeks, and exosomes loaded with RBP-J decoy or control decoy ODNs (exosomes/decoy ODNs = 200 μg/2.5 nmol) were injected into mice four times via tail vein at the last 2 weeks. F4/80^+^ hepatic macrophages were isolated using magnetic-activated cell sorting (MACS). The mRNA levels of Hes1, Hey1, TNFα, and IL1β in F4/80^+^ hepatic macrophages were measured by qRT-PCR. Bars = means ± SD; * P < 0.05.

Exosomes isolated from culture supernatants of a mouse endothelial cell line bEnd.3 to further reduce the immunogenicity of exosomes compared with HEK293T. As determined by Western blot, bEnd.3-derived exosomes had an enrichment of exosomal marker proteins CD9 and Alix, and contained a small amount of flotillin-1, whereas the expression of mitochondrial membrane protein VDAC1 was undetectable (Fig. 4A). NTA assay showed that the particle size of most bEnd.3-derived exosomes was below 200 nm, and electroporation had no significant effect on its particle size (Fig. 4B). Moreover, TEM indicated that the exosomes had a bilayer structure and electroporation has little effect on its morphology (Fig. 4C).

### Exosomes delivered via the tail vein were mainly taken up by hepatic macrophages and inhibited macrophage Notch signaling in a murine model of steatohepatitis

We tested the distribution of exosomes injected intravenously in steatohepatitis. We established a mouse model of steatohepatitis by feeding CDAA diet. Mice were infused with DiI-labeled exosomes (200 μg/mouse) via the tail vein. As expected, bioluminescence imaging analysis showed that DiI-labeled exosomes were mainly distributed in the liver 6 h after injection (Fig. 4D). Moreover, immunofluorescence staining of liver sections indicated DiI-labeled exosomes mainly located in F4/80^+^ macrophages 6 h after injection (Fig. 4E). These findings suggested that bEnd.3-derived exosomes were taken up by hepatic macrophages in mice with steatohepatitis.

**Figure 5:**
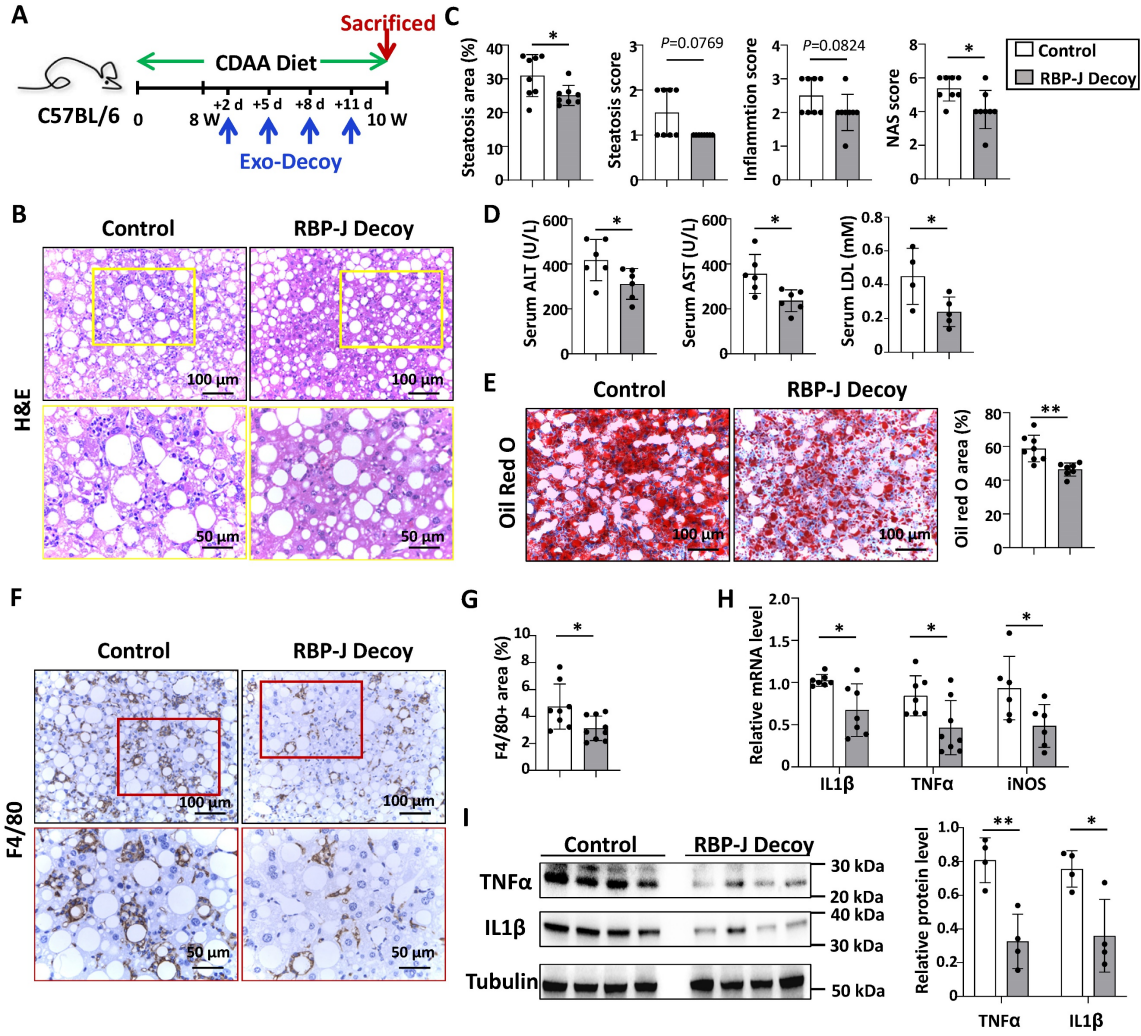
** Exosomes loaded with RBP-J decoy ODNs attenuated experimental steatohepatitis in CDAA-fed mice.** (A) Schematic illustration of the procedure used for exosomes loaded with RBP-J decoy ODNs or control decoy ODNs to treat steatohepatitis in mice fed with CDAA diet. (B) Liver sections were stained by H&E staining. The lower row of micrographs were a higher magnification of the yellow frames in the upper row. (C) Quantitative comparison of steatosis areas, and histological scores about steatosis, inflammation and NAS in (B). (D) Detection of ALT, AST and LDL in serum. (E) Liver sections were stained with Oil Red O, and areas positive were quantified and compared. (F) Liver sections were subjected to immunohistochemical staining for F4/80. The lower row of micrographs were a higher magnification of the red frames in the upper row. (G) The positive areas of F4/80 staining in (F) were quantitatively compared. (H) The mRNA levels of IL1β, TNFα and iNOS in livers were determined by qRT-PCR. (I) The protein levels of IL1β and TNFα were determined by Western blot, with Tubulin as a reference control. Bars = means ± SD, * P < 0.05, ** P < 0.01.

We next assessed if exosomes loaded with RBP-J ODNs were functional in hepatic macrophages. ODNs were loaded into bEnd.3-derived exosomes by electroporation, as previously reported 20, 23. CDAA-fed mice were injected with Exo-RBP-J decoy or Exo-control decoy ODNs via the tail vein (Fig. 5A). Hepatic F4/80^+^ cells were isolated using MACS as previously reported 20. As determined by qRT-PCR, the levels of Hes1, Hey1, IL1β and TNFα were downregulated in hepatic F4/80^+^ cells derived from Exo-RBP-J decoy ODN-treated mice compared with mice treated with Exo-control decoy ODNs (Fig. 4F). These results suggested that exosomes loaded with RBP-J decoy ODNs could inhibit the activation of Notch signaling in hepatic macrophages *in vivo*.

### Exo-RBP-J decoy ODNs attenuated experimental steatohepatitis in CDAA-fed or steatosis in HFD-fed mice

Next, we evaluated the efficacy of Exo-RBP-J decoy ODNs in CDAA-fed or HFD-fed mice. As shown in Fig. 5A, mice were fed with CDAA diet for 10 weeks, and infused four times with Exo-RBP-J decoy or Exo-control decoy ODNs (200 μg/mouse) via tail vein during the last 2 weeks. H&E staining of liver sections showed that the large round non-staining areas and infiltrating cells were less in Exo-RBP-J decoy ODN-treated group compared with control (Fig. 5B,5C). Meanwhile, liver sections were assessed with NAS score. The results indicated that NAS score in Exo-RBP-J decoy ODN-treated mice were lower than control mice (Fig. 5C). Accordingly, Oil Red O staining of liver sections also showed that Exo-RBP-J decoy ODN-treated mice had less lipid accumulation in livers compared with control mice (Fig. 5E). Immunostaining with anti-F4/80 and anti-MPO showed that the infiltration of macrophages and neutrophils was less in livers from Exo-RBP-J decoy ODN-treated mice (Fig. 5F.5G, Fig. S7). Accordingly, the levels of inflammatory mediators were determined using qRT-PCR and Western blot. The results indicated that IL1β, TNFα and iNOS were decreased in the livers from mice treated with Exo-RBP-J decoy ODNs (Fig. 5H.5I). The level of IL1β in the serum also decreased in Exo-RBP-J decoy ODNs treated mice as compared with the control mice (Fig. S3B). In keeping with this, serum ALT, AST and LDL levels were decreased in Exo-Decoy RBP-J ODN recipient mice, suggesting that liver function had improved (Fig. 5D). Then we evaluated hepatic fibrosis by anti-αSMA, anti-Col1α immunohistochemical staining and Sirius red staining. Exo-RBP-J decoy ODN-treated mice displayed significantly decreased activation of hepatic stellate cells and ECM deposition (Fig. S8A,B). Consistently, the mRNA levels of platelet derived growth factor subunit B (PDGF-B), TIMP2, Col1α1 and αSMA decreased in the livers of Exo-RBP-J decoy ODN-treated mice (Fig. S8C). These results indicated that Exo-RBP-J decoy ODNs ameliorated steatohepatitis and steatohepatitis-induced fibrosis in CDAA-fed mice.

**Figure 6:**
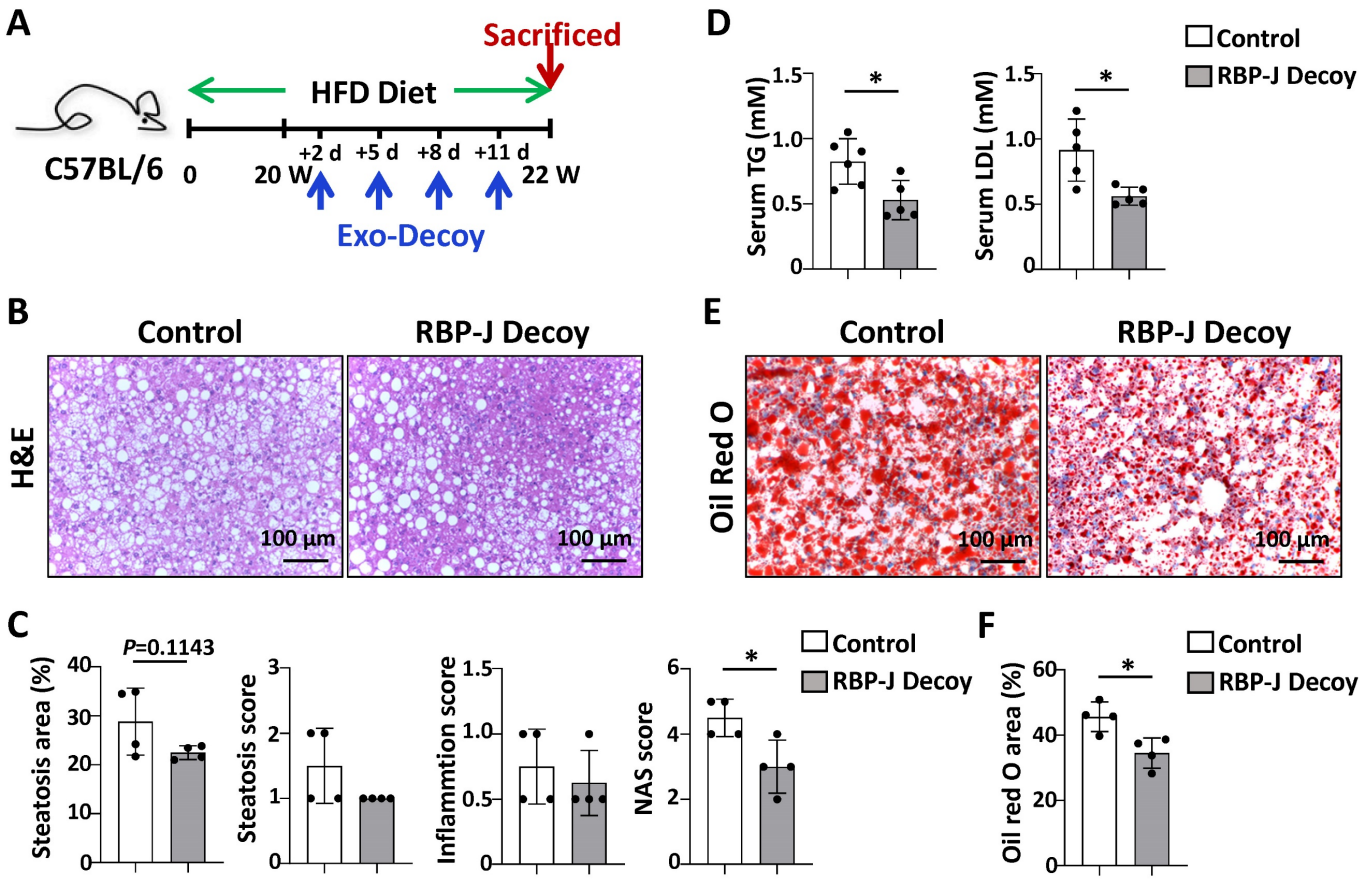
** Exosomes loaded with RBP-J decoy ODNs attenuated fatty liver in HFD-fed mice.** (A) Schematic illustration of the procedure used for exosomes loaded with RBP-J decoy ODNs or control decoy ODNs to treat steatosis in HFD diet-fed mice. Mice were fed with HFD diet for 22 weeks, and exosomes loaded with RBP-J decoy or control decoy ODNs (exosomes/decoy ODNs = 200 μg/2.5 nmol) were injected into mice four times via tail vein at the last 2 weeks. (B) Liver sections were stained by H&E staining. (C) Quantitative comparison of steatosis areas, and histological scores about steatosis, inflammation and NAS in (B). (D) Detection of triglyceride content and LDL in serum. (E) Liver sections were stained with Oil Red O, and areas positive were quantified and compared in (F). Bars = means ± SD; * P < 0.05.

To confirm the effect of Exo-RBP-J decoy ODNs in NAFLD, the experiment was repeated using another mouse model, HFD-induced steatosis. As shown in Fig 6A, mice were fed with HFD diet for 22 weeks, and infused four times with Exo-RBP-J decoy or Exo-control decoy ODNs via tail vein during the last 2 weeks. Histological examination of liver sections using with H&E or oil red O staining showed that Exo-RBP-J decoy ODNs-treated mice had less lipid accumulation in livers compared with control mice (Fig. 6B,C,E,F). Meanwhile, NAS score was also decreased in the livers of Exo-RBP-J decoy ODN-treated mice (Fig. 6C). Accordingly, triglyceride content and LDL in serum were significantly decreased in Exo-RBP-J decoy ODNs-treated mice (Fig. 6D). Overall, these data suggested that Exo-RBP-J decoy ODNs application could attenuate experimental steatohepatitis in CDAA-fed or steatosis in HFD-fed mice.

## Discussion

Studies have shown that Notch signaling can regulate key processes of in liver development, homeostasis, and disease 36-38. Pajvani's group demonstrated that hepatocyte Notch signaling was activated in patients with NASH and a mouse model of diet-induced NASH 39,40. They further developed a poly(lactic coglycolic acid) (PLGA)-encapsulated γ-secretase inhibitor nanoparticles (GSI NPs) to target hepatocytes, and found that GSI NPs application ameliorated liver inflammation and fibrosis in NASH diet-fed mice 41.

Interestingly, in this study we found that Notch signaling was activated in hepatic macrophages in human with NASH or in CDAA-fed mice. Several groups have reported that Notch signaling regulates macrophage activation through multiple downstream molecules, such as IRF8, SOCS3, Cyld, miR-125a, miR-148a and signal regulatory protein α (SIRPα) 15-19,42-44. Therefore, we speculated that macrophage Notch signaling may play a role in the development of NAFLD. We then established a CDAA-induced steatohepatitis model in Lyz2-Cre RBP-J^flox^ mice and found that myeloid-specific RBP-J deficiency attenuated liver inflammation, lipid accumulation and hepatic fibrosis. Similar to our previous results in CCl_4_-induced hepatic fibrosis, the expression of inflammatory factors IL1β and TNFα was significantly decreased in the livers of myeloid-specific RBP-J knockout mice with NAFLD in this study. As we known, macrophage-derived inflammatory mediators may influence hepatocyte steatosis. Stienstra et al. 24 reported that hepatic macrophages promoted hepatic steatosis via IL1β-dependent suppression of Pparα) activity. Miura et al. 25 showed that IL1β derived from hepatic macrophages increased lipid accumulation in hepatocytes. Zhang et al. 12 reported that macrophage p38a could promote lipid accumulation in hepatocytes through the induction of cytokines TNFα, CXCL10 and IL6. Diehl et al. 26 also showed that TNFα secretion by hepatic macrophages was necessary and sufficient to induce steatosis in hepatocytes *in vitro*. And our results indicated that CM derived from macrophages treated with GSI reduced lipid accumulation in hepatocytes, but this capacity was rescued partially by supplement of IL1β or TNFα, and recovered completely by supplement of IL1β and TNFα.

To explain how pro-inflammatory cytokines IL1β and TNFα affect lipid accumulation in hepatocytes, we examined the expression of classical genes associated with fatty acid metabolism in the liver samples of CDAA-fed RBP-J KO and control mice. However, we didn't obtain differentially expressed genes except Pparɑ. Therefore, we compared mRNA profiles of liver tissues between CDAA-fed RBP-J KO and control mice by using RNA-seq. We found that the expression of Pparɑ, Cyp4a12a/b, Acaa1a, Nudt7 and Pex11ɑ was up-regulated in RBP-J knockout group. Interestingly, these genes, which are regulated by Pparɑ, are closely related to peroxisomal fatty acid oxidation and fatty acid degradation 27-34. Naturally, we speculated that macrophage RBP-J knockout can attenuate the expression of TNFɑ and IL1β, then the reduced TNFɑ and IL1β may enhance the expression or activity of Pparɑ in hepatocytes, up-regulate the expression of Cyp4a12a/b, Acaa1a and Pex11ɑ, promote peroxisomal fatty acids oxidation and fatty acid degradation, and finally reduce lipid accumulation in hepatocytes. This hypothesis deserves further study.

We then sought to determine the feasibility of treating NAFLD by inhibiting Notch signaling in macrophages. Several key points need to be weighed carefully. First, selecting a proper drug delivery system to target hepatic macrophages. Exosomes are nano-sized phospholipid bilayer-enclosed vesicles secreted by living cells and have the advantage of lower immunogenicity compared with exogenous nanocarriers 45, 46. Importantly, exosomes injected intravenously were mainly distributed in the liver and taken up by hepatic macrophages 47, 48. This indicates that exosomes may represent a natural delivery system for the targeting of hepatic macrophages. In this study, we further demonstrated that exosomes injected via tail vein were taken up by hepatic macrophages in CDAA-fed mice.

Second, selecting appropriate Notch inhibitor. Transcription factor decoy (TFD) ODNs harboring the consensus DNA recognition motif of a target transcription factor can compete with the binding sites in the promoter regions of target genes for the binding of transcription factor, and significantly reduce the expression levels of downstream genes 49. Several studies have investigated the application of NF-κB decoy ODNs in the treatment of inflammatory disorders 50,51. We previously have systematically demonstrated that RBP-J decoy ODNs can inhibit the activation of Notch signaling in macrophages 20.

Third, we need to evaluate the efficiency for ODNs loading into exosomes. As previously reported, the efficiency of electroporation was about 9% 20. Exosomes can also be loaded with drugs by other methods, such as co-incubation, ultrasound treatment, saponin treatment, density gradient ultracentrifugation, and freeze-thaw extrusion 52. We will compare the drug delivery efficiency of other methods in the future work.

Finally, we need to confirm that exosomes loaded with RBP-J decoy ODNs could work after entering macrophages. Exosomes could be internalized into mammalian cells mainly by two different mechanisms including direct membrane fusion and endocytosis 53. Parolini et al. 54 and Montecalvo et al. 55. verified that the cargoes would release to the cytosol once exosomes have fused with membrane. On the other hand, exosomes enter cells through the endocytosis pathway 53. To complete cargo delivery, exosomes need to escape the endo/lysosomal membrane, a process is known as “endosomal escape”. Although there is currently no direct evidence that exosomes can escape the endo/lysosome, numerous studies have claimed that exosomes can functionally deliver cargo to recipient cells and induce substantial phenotypic changes 56-60. As for our study, we sorted hepatic macrophages from CDAA mice, detected the expression of Notch downstream genes, and functionally verified the inhibitory effects of RBP-J decoy ODNs *in vivo*.

Studies have shown that hepatic macrophages are a heterogeneous population of immune cells, consisted of resident macrophages (Kupffer cells) and monocyte-derived macrophages. Kupffer cells are established during embryonic development from fetal yolk-sack, whereas monocyte-derived macrophages originate from circulating monocytes 6-8. The RBP-J gene of Kupffer cells or monocyte-derived macrophages was deficient in Lyz2-Cre RBP-J^flox/flox^ transgenic mice (data not shown). What are the effects of different macrophage populations on NAFLD? Does Notch signaling play a role among them? This deserves further study.

In summary, we demonstrated that Notch signaling was activated in hepatic macrophages in human with NASH or in mice with experimental steatohepatitis. We found that myeloid-specific RBP-J deficiency could attenuate experimental steatohepatitis in mice. One of the mechanisms may be that Notch signaling blockade attenuates the expression of inflammatory cytokines IL1β and TNFα in macrophages, thereby inhibits lipid accumulation in hepatocytes by enhancing peroxisomal fatty acid oxidation. Furthermore, our results showed that injection of exosomes loaded with RBP-J decoy ODNs via tail veil could efficiently inhibit Notch signaling in hepatic macrophages, and ameliorate NAFLD in mice. Thus, infusion of exosomes loaded with RBP-J decoy ODNs may represent a promising therapeutic strategy for the treatment of NAFLD that merits further investigation.

## Supplementary Material

Supplementary figures and tables.

## Figures and Tables

**Figure 7:**
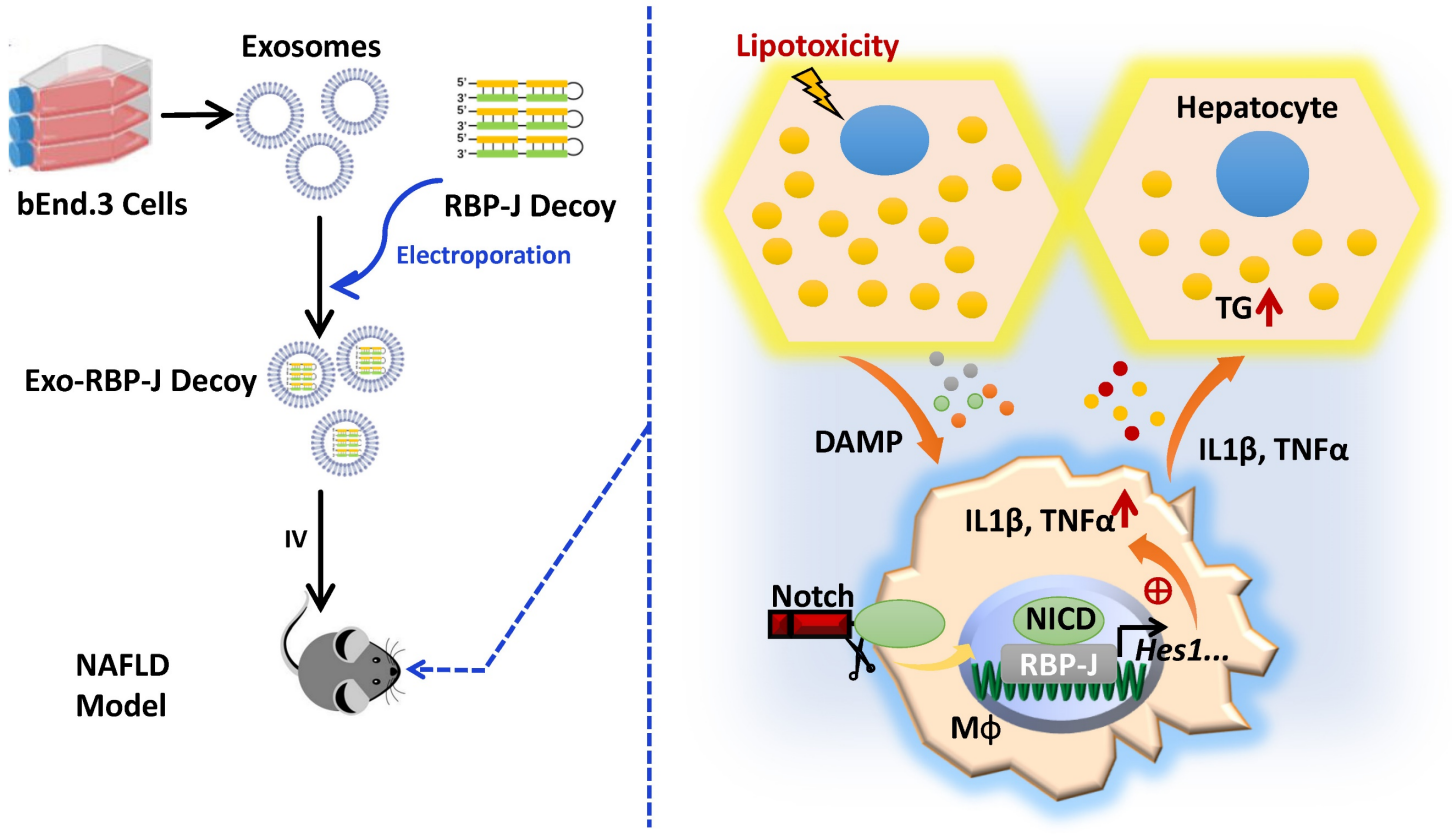
** Schematic summary of this study.** During the progression of NAFLD, injured hepatocytes caused by over-accumulated lipid toxicity, released damage-associated molecular patterns (DAMPs), and then stimulated hepatic macrophages to release pro-inflammatory cytokines such as IL1β, TNFα. Meanwhile, Notch signaling, mediated by transcription factor RBP-J, was activated in hepatic macrophages and further promoted the expression of IL1β and TNFα. And then IL1β and TNFα in turn enhanced lipid accumulation in hepatocytes. Next, we used exosomes targeting delivery of RBP-J decoy ODNs and inhibited the activation of Notch signaling in macrophages. Finally, the infused exosomes-RBP-J decoy ODNs via tail vein resulted in the amelioration of NAFLD in mice.
