# Supplementary material for: Myeloid-specific blockade of Notch signaling ameliorates nonalcoholic fatty liver disease in mice
Source: Int J Biol Sci. 2023 Mar 27;19(6):1941–54. doi: 10.7150/ijbs.80122 (PMC10092768; doi:10.7150/ijbs.80122)
Supplement: Supplementary file 1 — Supplementary figures and tables. [file ijbsv19p1941s1.pdf]

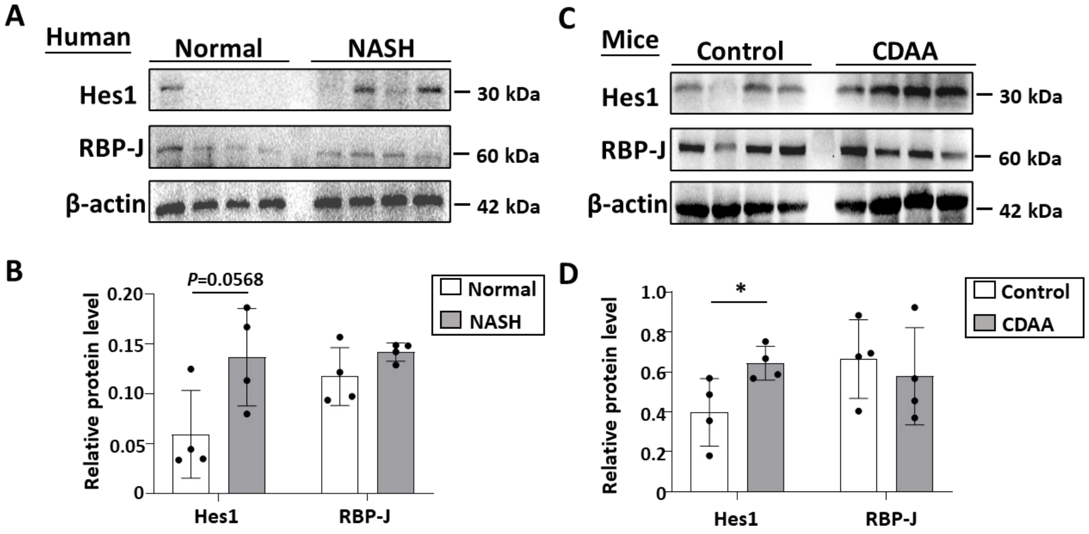

Fig.S1 The Hes1 expression of the livers was up-regulated in human with NASH or in CDAA-fed mice. (A) The protein levels of Hes1 and RBP-J in liver extracts from normal human or NASH patients were determined by Western blot, with β-actin as a reference control. (B) The images in (A) was imported into Image J and the gray value of the bands was quantitatively analyzed. (C) The protein levels of Hes1 and RBP-J in liver extracts from chow-fed or CDAA-fed mice were determined by Western blot. (D) The images in (C) were quantitatively analyzed. Bars = means ± SD, * $P < 0.05$.

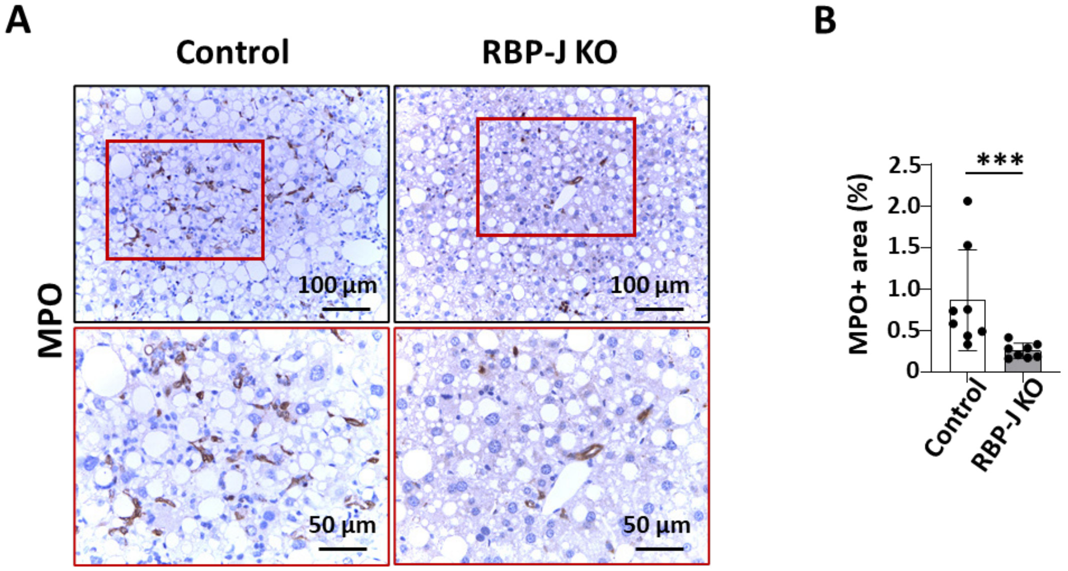

Fig.S2 Macrophage-specific RBP-J deficiency reduced neutrophil infiltration in the liver samples of CDAA mice.

(A) Liver sections were subjected to immunohistochemistry staining with anti-MPO antibody. The lower row of micrographs were a higher magnification of the red frames in the upper row. (B) Quantitative comparison of positive signals in (A). Bars = means ± SD, *** $P < 0.001$.

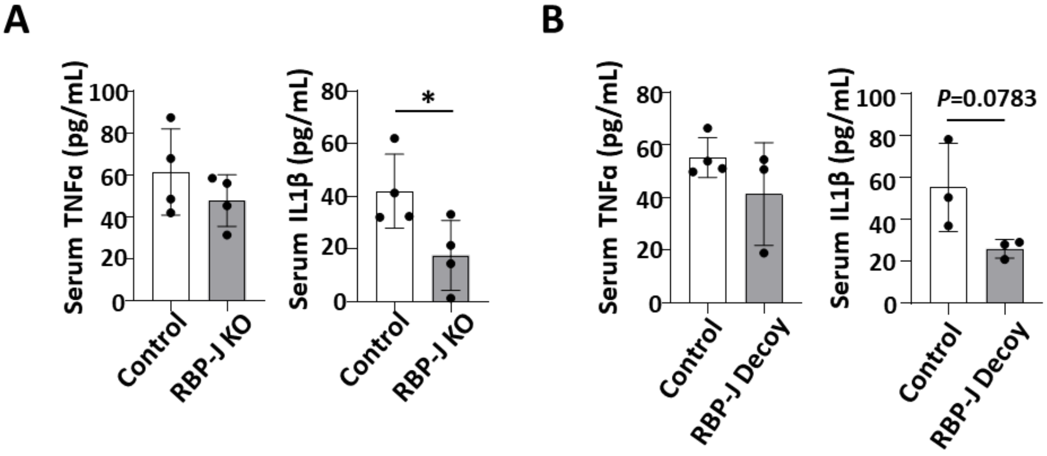

Fig.S3 Notch blockade in macrophages reduced the levels of IL1β in the serum. (A) Lyz2-Cre^{+} RBP-J$^{flox/+}$ (Control) or Lyz2-Cre^{+} RBP-J$^{flox/flox}$ (KO) mice were fed with CDAA diet for 10 weeks. The levels of TNFα and IL1β in the serum were analyzed by ELISA. (B) CDAA-fed mice were treated with Exo-Decoy RBP-J or Exo-Decoy control ODNs. Serum TNFα and IL1β were detected by using ELISA. Bars = means ± SD, * $P < 0.05$.

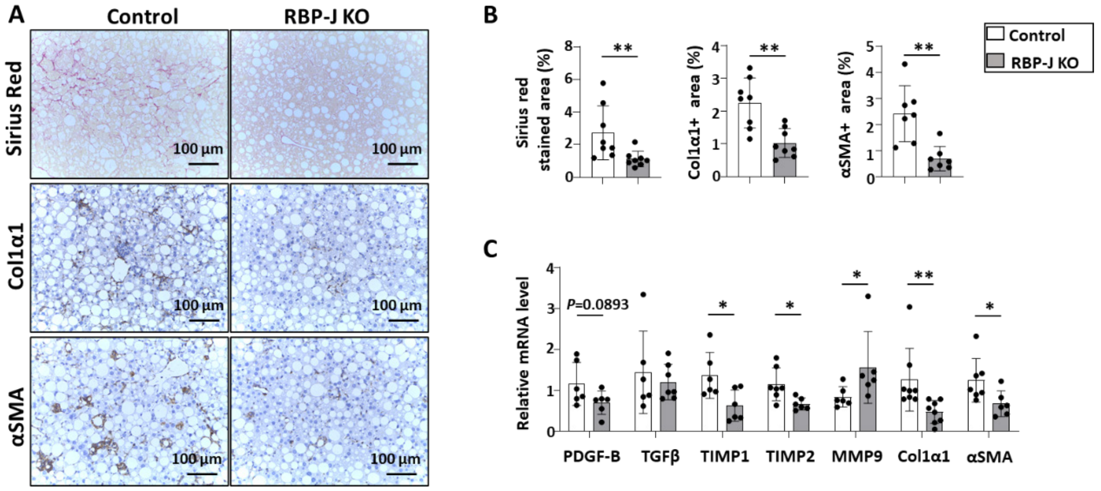

Fig.S4 Macrophage-specific RBP-J deficiency attenuated experimental steatohepatitis-induced hepatic fibrosis in CDAA-fed mice.

(A) Liver sections were stained with Sirius Red, or immunohistochemistry with anti-Col1α or anti-αSMA. (B) Positive signals for Sirius Red or immunohistochemistry staining were quantitatively compared. (C) The mRNA levels of hepatic fibrosis associated genes PDGF-B, TGFβ, TIMP1, TIMP2, MMP9, Col1α1 and αSMA were determined by qRT-PCR. Bars = means ± SD, * $P < 0.05$, ** $P < 0.01$.

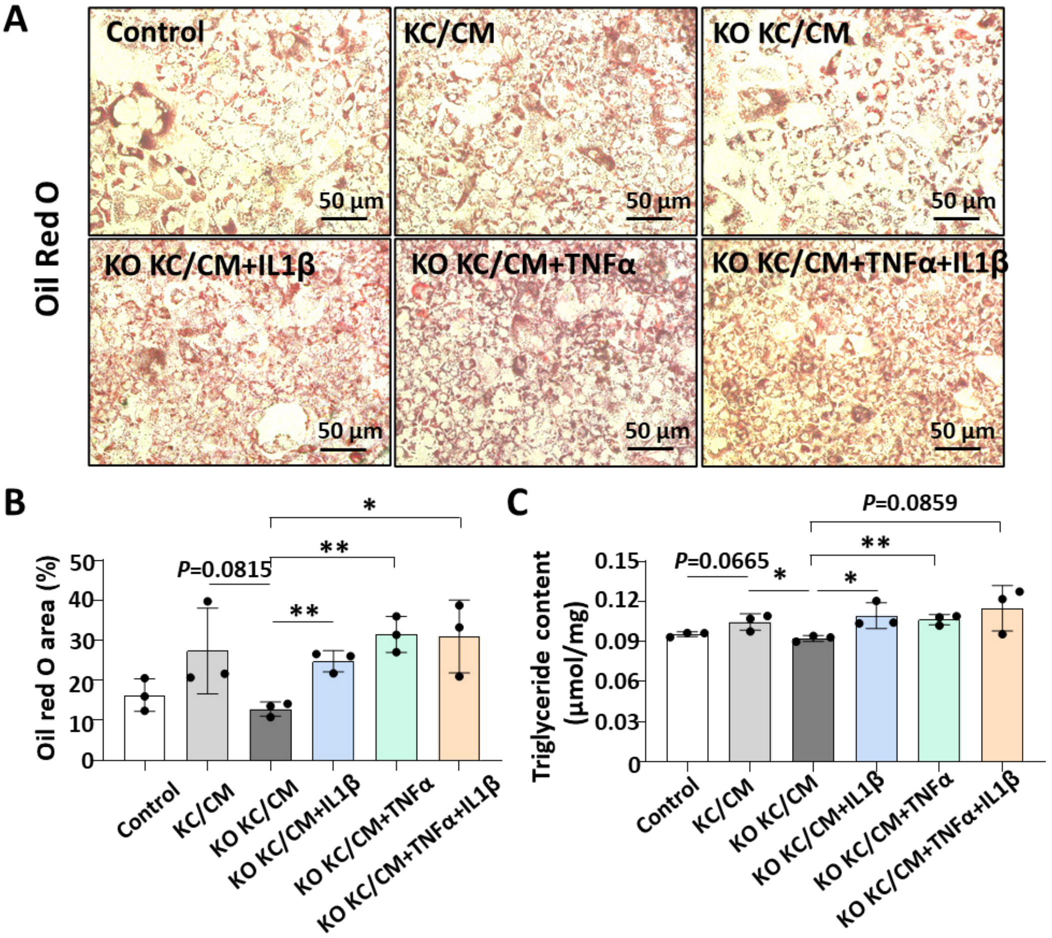

Fig.S5 RBP-J deficiency in hepatic macrophages attenuated lipid accumulation in hepatocytes through inhibiting the expression of IL1β and TNFα *in vitro*. Hepatic macrophages were isolated and cultured with the fresh medium containing LPS (100 ng/ml) for 1 day, and the conditioned medium (CM) were harvested. AML12 hepatocytes were cultured with CM, in the presence or absence of TNFα (10 ng/ml), and/or IL1β (10 ng/ml) in palmitic acid medium (PA, 10 mM) for 2 days. (A) Lipid accumulation in AML12 cells was assessed with oil red O staining. (B) The positive areas of oil red O staining in (A) were quantitatively compared. (C) Triglyceride content in AML12 hepatocytes was measured. Bars = means ± SD; * $P < 0.05$, ** $P < 0.01$.

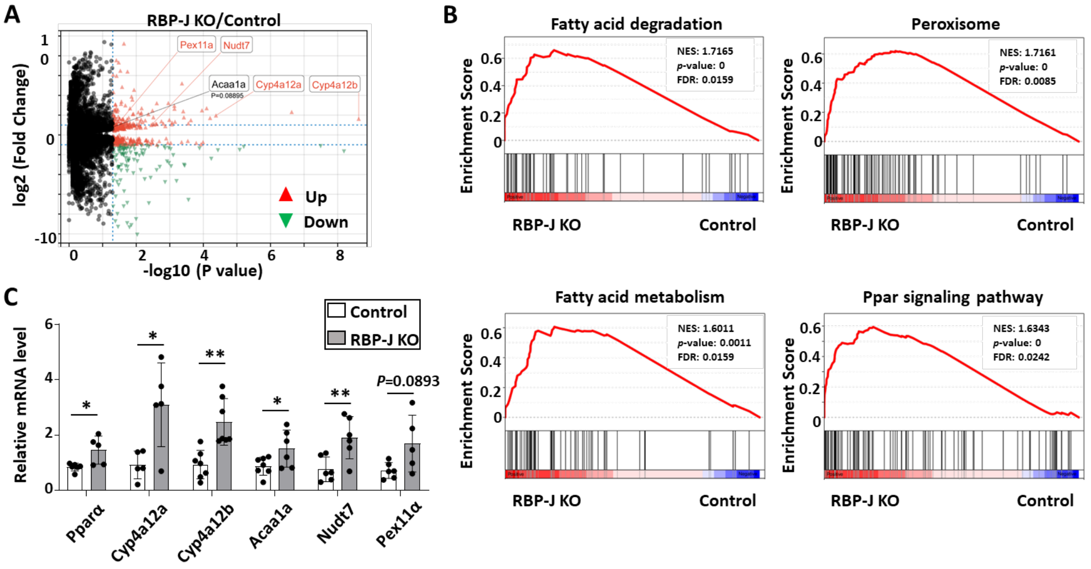

Fig.S6 Macrophage-specific RBP-J deficiency up-regulated the expression of genes related to fatty acid degradation and peroxisomal fatty acid oxidation in livers of CDAA-fed mice. Lyz2-Cre^{+} RBP-J$^{flox/+}$ (Control) or Lyz2-Cre^{+} RBP-J$^{flox/flox}$ (RBP-J KO) mice were fed with CDAA diet for 10 weeks. The mRNA expression of liver samples was profiled by using RNA-seq (n = 4). (A) The volcano plot showed the differentially expressed genes between RBP-J KO and control groups. (B) Gene set enrichment analyses of RBP-J KO and control groups. The enrichment of fatty acid degradation, peroxisome, fatty acid metabolism and Ppar signaling pathway were investigated. (C) Expression of Pparα, Cyp4a12a, Cyp4a12b, Acaa1a, Nudt7, and Pex11α were determined by qRT-PCR. Bars = means ± SD; * $P < 0.05$, ** $P < 0.01$.

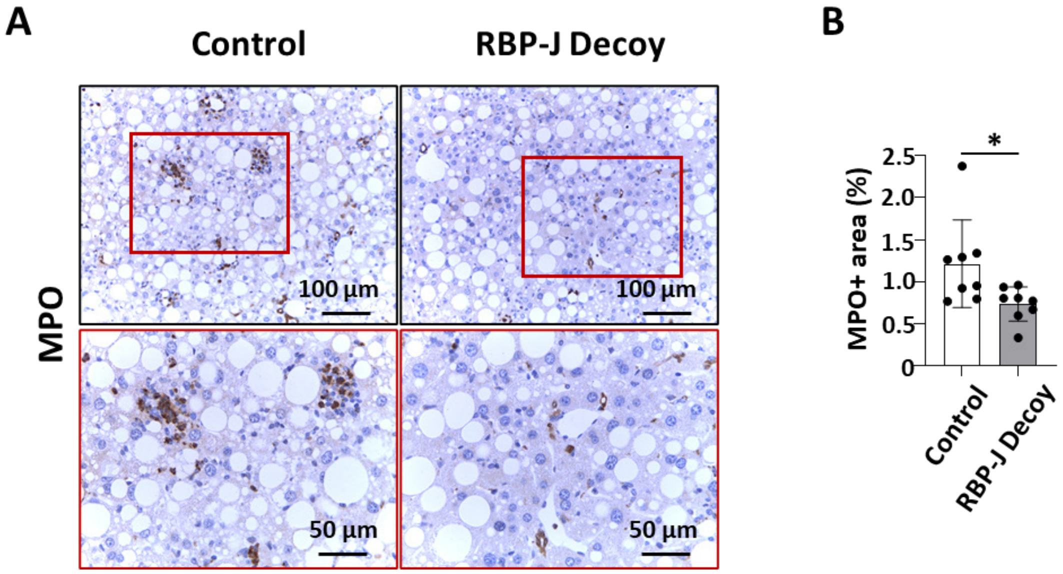

Fig.S7 Exosomes loaded with RBP-J decoy ODNs reduced neutrophil infiltration in the liver of CDAA mice.

(A) Liver sections were subjected to immunohistochemistry staining with anti-MPO antibody. The lower row of micrographs were a higher magnification of the red frames in the upper row. (B) Quantitative comparison of positive signals in (A). Bars = means ± SD, * $P < 0.05$.

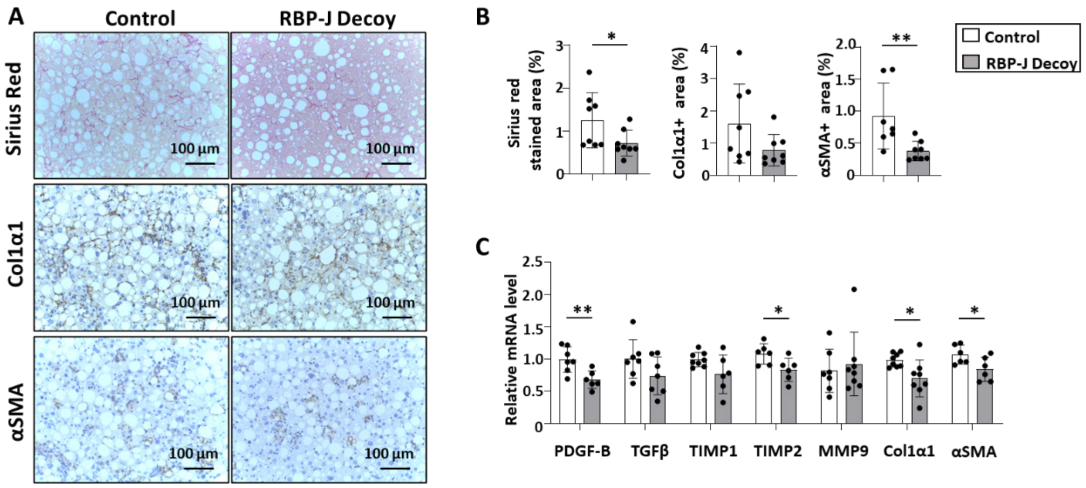

Fig.S8 Exosomes loaded with RBP-J decoy ODNs ameliorated experimental steatohepatitis-induced hepatic fibrosis in CDAA-fed mice.

(A) Liver sections were subjected to Sirius Red staining, or immunohistochemistry staining with anti-Col1α or anti-αSMA. (B) Quantitative comparison of positive signals in (A). (C) The mRNA levels of liver fibrosis associated genes PDGF-B, TGFβ, TIMP1, TIMP2, MMP9, Col1α1 and αSMA were determined by qRT-PCR. Bars = means ± SD, * $P < 0.05$, ** $P < 0.01$.

Table S1. Basic information of patients.

Normal				NASH			
Number	Gender	Age	BMI	Number	Gender	Age	BMI
1	Female	36	21.7	1	Female	38	34.9
2	Female	33	25.2	2	Female	35	46.2
3	Female	71	22.1	3	Male	67	23.6
4	Male	26	21.7	4	Male	46	25.9
5	Male	70	19.5	5	Female	71	29.0
6	Male	58	22.4	6	Female	54	23.6
				7	Female	54	24.5

Table S2. Antibodies used in this study

Antibody	Supplier	Cat.No	Purpose
Hes1	Cell Signaling Technology	D6P2U	IF
CD68	Abcam	Ab955	IF
α-SMA	Servicebio	GB111364	IHC
Col1α1	Servicebio	GB11022-3	IHC
MPO	Servicebio	GB11224	IHC
F4/80	Invitrogen	14-4801-82	IF
Tubulin	Proteintech	10068-1-AP	WB
Goat anti-Rabbit-HRP	Abbkine	21020	WB
Goat anti Rabbit Cy3	Jackson Immuno Research	111-165-003	IF
Goat anti Rabbit FITC	Jackson Immuno Research	111-095-003	IF
IL1β	Proteintech	26048-1-AP	WB
TNFα	Proteintech	17590-1-AP	WB
CD9	Cell Signaling Technology	98327	WB
Alix	Cell Signaling Technology	92880	WB
Flotillin-1	Cell Signaling Technology	18634	WB
VDAC-1	Cell Signaling Technology	4661	WB
β-actin	Proteintech	81115-1-RR	WB

Table S3. Sequences of primers used in the study

Gene	Forward(5’-3’)	Reverse(5’-3’)
β-actin	GGCTGTATTCCCCTCCATCG	CCAGTTGGTAACAATGCCATG
GAPDH	AGGTCGGTGTGAACGGATTTG	TGTAGACCATGTAGTTGAGGTCA
Notch1	GATGGCCTCAATGGGTACAAG	ACATATCGAGATTGGGGTGTCT
Notch2	CGCAGGTTCTTGGTCACTGT	TGTTCACGAAAGCCAGAGCG
Notch3	CCTGGTGATGTCCGACCTG	CCATGAGCGCATCGCAATC
Notch4	TTAAAAACCTGGATCGGAACCAA	GCATTAGCTTCAGATTTACGGGT
Jag1	CCTCGGGTCAGTTTGAGCTG	CCTTGAGGCACACTTTGAAGTA
Jag2	CTGTGCAGCGTGTTCAGTG	GTGTCCACCATACGCAGATAAC
Dll1	CAGGACCTTCTTTCGCGTATG	AAGGGGAATCGGATGGGGTT
Dll4	TTCCAGGCAACCTTCTCCGA	ACTGCCGCTATTCTTGTCCC
Hes1	TCAGCGAGTGCATGAACGAG	CATGGCGTTGATCTGGGTCA
Hey1	CCGACGAGAGACCGAATCAATA	TCAGGTGATCCGAATCAATA
IL1β	GAAATGCCACCTTTTGACAGTG	TGGATGCTCTCATCAGGACAG
TNFα	CCCTCACACTCAGATCATCTTCT	GCTACGACGTGGGCTACAG
iNOS	GCAGAGATTGGAGGCCTTGTG	GGGTTGTTGCTGAACTTCCAGTC
PDGF-B	TACCTGCGTCTGGTCAGC	GCTCGGGTCATGTTCAAG
TGF-β	CTTCGACGTGACAGACGCT	GCAGGGGCAGTGTAAACTTATT
TIMP1	CGAGACCACCTTATACCAGCG	ATGACTGGGGTGTAGGCGTA
TIMP2	TCAGAGCCAAAGCAGTGAGC	GCCGTGTAGATAAACTCGATGTC
MMP9	CTGGACAGCCAGACACTAAAG	CTCGCGGCAAGTCTTCAGAG
Col1α	GCTCCTCTTAGGGGCCACT	CCACGTCTCACCATTGGGG
αSMA	CCCAGACATCAGGGAGTAATGG	TCTATCGGATACTTCAGCGTCA
Pparα	AGAGCCCCATCTGTCCTCTC	ACTGGTAGTCTGCAAAACCAAA
Cyp4a12a	CCTCTAATGGCTGCAAGGCTA	CCAGGTGATAGAAGTCCCATCT
Cyp4a12b	GGGGAGATCAGACCCAAAAGC	ATTCGTCGGTGCTGAAACCAT
Acaa1a	TCTCCAGGACGTGAGGCTAAA	CGCTCAGAAATTGGGCGATG
Nudt7	AAGGCTCGCCTGAGAAAGTC	GTATGGCACCAGGTGAGAGA
Pex11α	GACGCCTTCATCCGAGTCG	CGGCCTCTTTGTCAGCTTTAGA
